# Extracellular Vesicles in Myocardial Infarction: Dual Role in Ferroptosis Regulation and In Vivo Imaging

**DOI:** 10.3390/diagnostics16091305

**Published:** 2026-04-27

**Authors:** Cong Zhang, Yang Hou

**Affiliations:** Department of Radiology, Shengjing Hospital of China Medical University, Shenyang 110055, China; 13236984111@163.com

**Keywords:** ferroptosis, myocardial infarction, extracellular vesicle, molecular imaging, multimodal imaging

## Abstract

Acute myocardial infarction (AMI), a life-threatening event caused by cardiomyocyte death due to oxygen deprivation, drives cardiac dysfunction through ferroptosis—an iron-dependent cell death mechanism involving lipid peroxidation. By delivering multifunctional cargoes with low immunogenicity, extracellular vesicles (EVs) hold the therapeutic potential to inhibit cardiomyocyte ferroptosis through the regulation of iron metabolism and the mitigation of oxidative damage. Their dual role as targeted drug carriers and natural imaging probes enhances precision in AMI management. EVs enable the non-invasive tracking of biodistribution and therapeutic responses in real time when integrated with molecular imaging technology, offering insights into cardiac repair mechanisms. This synergy between EV-based therapy and advanced imaging presents a novel strategy for AMI diagnosis and targeted intervention.

## 1. Introduction

AMI remains a leading cause of global mortality. It involves cardiomyocyte death due to ischemia, with ferroptosis—an iron-dependent form of programmed cell death driven by lipid peroxidation—gaining recognition as a key pathogenic mechanism [1]. Ferroptosis arises from dysregulated iron, lipid, and amino acid metabolism, leading to glutathione peroxidase inactivation and toxic lipid hydroperoxide accumulation [2]. Therapeutic strategies such as antioxidants and iron chelators show promise in mitigating ferroptosis [3,4].

Extracellular vesicles (EVs) are broadly categorized into three main types based on their biogenesis: exosomes (approximately 30–150 nm in diameter, formed via endosomal pathways, carrying bioactive cargo and mediating intercellular communication), which are endosome-derived vesicles released upon the fusion of multivesicular bodies with the plasma membrane; microvesicles (also known as ectosomes, approximately 100–1000 nm in diameter), which are generated by direct outward budding of the plasma membrane; and apoptotic bodies (approximately 500–5000 nm in diameter), which are released from cells undergoing apoptosis. In cardiovascular disease, EVs serve as diagnostic biomarkers and therapeutic vectors due to their low immunogenicity and tissue-penetrating ability [5]. They modulate ferroptosis in cardiomyocytes by transferring signaling molecules, influencing AMI progression. Advances in molecular imaging enable in vivo tracking of EVs, offering novel insights into AMI pathophysiology and treatment [6].

## 2. The Mechanism of Ferroptosis in Acute Myocardial Infarction

**Figure 1:**
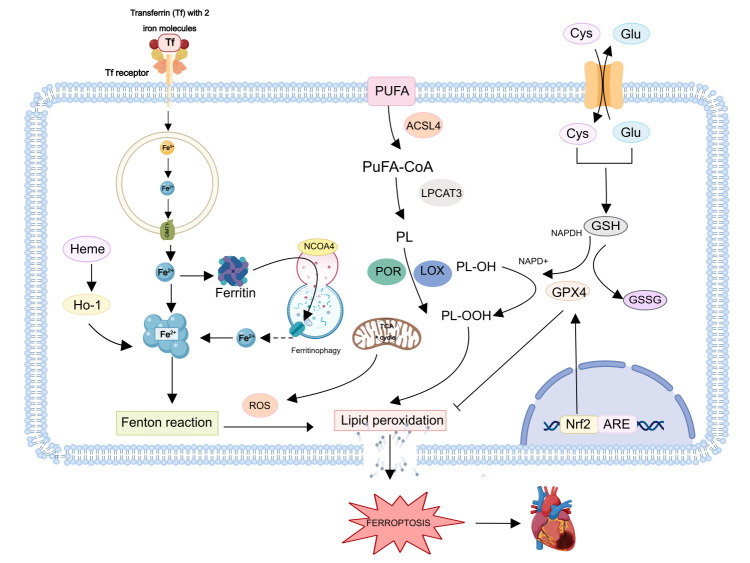
Ferroptosis is an iron-dependent regulated cell death process governed by three core axes: iron dyshomeostasis, lipid peroxidation, and antioxidant defense. Iron metabolism begins with TFR1-mediated endocytosis of iron-transferrin complexes. In endosomes, STEAP3 reduces Fe^3+^ to Fe^2+^, which is transported into the labile iron pool (LIP) by DMT1. Fe^2+^ drives Fenton reactions, generating ·OH radicals that initiate PUFA-PL peroxidation. Lipid metabolism involves ACSL4, and LPCAT3, which incorporate PUFA into membranes, while Lipoxygenases (LOXs) catalyze lipid peroxide formation. Amino acid metabolism relies on System Xc^−^ and GPX4 to maintain redox balance, with NRF2 upregulating GPX4 to bolster antioxidant defenses. Conversely, NCOA4-mediated ferritinophagy liberates stored iron, forming a feedforward loop that amplifies ferroptosis, ultimately leading to membrane disintegration and cell death.

Ferroptosis is an iron-dependent form of regulated cell death driven by lipid peroxidation, emerging as a critical pathogenic mechanism in Acute Myocardial Infarction (AMI). While iron is physiologically essential, its pathological accumulation in the heart disrupts redox homeostasis. Excessive cardiac iron triggers reactive oxygen species (ROS) generation via the Fenton reaction, exacerbating lipid peroxidation and myocardial injury. This iron-mediated oxidative damage positions ferroptosis as a key therapeutic target in AMI, with current research focusing on dysregulated iron homeostasis in ischemic cardiomyocyte death [7] (Figure 1).

### 2.1. Iron Metabolism

Iron is essential for human metabolism, but its dysregulation is central to ferroptosis pathogenesis in AMI. In cardiomyocytes, iron is imported via transferrin receptor 1 (TFR1)-mediated endocytosis, reduced to Fe^2+^ by six-transmembrane epithelial antigen of the prostate (STEAP) family metalloreductases, and transported into the cytoplasm by divalent metal transporter 1 (DMT1) [8]. Redox-active Fe^2+^ drives Fenton chemistry and lipoxygenase activation, initiating lipid peroxidation. Cellular iron homeostasis is regulated by two complementary mechanisms: ferritin-mediated storage and ferroportin-mediated export. Ferritin sequesters excess cytosolic iron into its protein cage, representing “transport to storage” for detoxification and iron reserve formation. In contrast, ferroportin exports intracellular Fe^2+^ to the extracellular space, mediating iron “clearance” for systemic distribution, primarily to support erythropoiesis. These processes are integrated via the hepcidin–ferroportin axis: hepcidin binding induces ferroportin degradation, reducing iron export and systemic iron availability, while ferritin responds primarily to local intracellular iron levels. Thus, ferritin governs intracellular iron sequestration, whereas ferroportin controls cellular iron clearance into the circulation [9].

In AMI, reperfusion injury and hemorrhage exacerbate iron deposition. Iron overload promotes macrophage pro-inflammatory polarization, foam cell formation, and adverse cardiac remodeling [10]. Clinical data link iron deposition with lipid accumulation and cardiac dysfunction in infarcted myocardium. Apoptotic foam cells release iron and lipids, perpetuating inflammation and injury [11].

Ferroptosis also releases Damage-Associated Molecular Patterns (DAMPs), which amplify inflammation and create a feed-forward loop [12]. Paradoxically, ischemic preconditioning (IPC) activates protective iron handling: Iron Regulatory Proteins (IRPs) enhance ferritin translation, increasing iron-chelating capacity and reducing iron-catalyzed radical generation during ischemia [13].

Quantitative susceptibility mapping (QSM) is an MRI technique based on the measurement of tissue magnetic susceptibility, which has shown significant application potential in cardiovascular imaging in recent years [14]. Due to the presence of multiple unpaired electrons, iron ions (Fe^2+^/Fe^3+^) exhibit strong paramagnetic properties, enabling QSM to directly detect their deposition in tissues with high specificity for iron. This approach offers advantages over conventional T_2_, T_2_*, or T_1_ mapping methods, which are susceptible to confounding factors such as edema, adipose tissue, and collagen [15].

Following AMI, intramyocardial hemorrhage (IMH) can lead to local iron deposition, thereby exacerbating myocardial injury and adverse remodeling. Numerous studies have demonstrated the efficacy of QSM in detecting and quantifying myocardial iron deposition post-MI. For instance, in a study involving patients with ST-segment elevation myocardial infarction (STEMI), QSM images clearly revealed localized increases in magnetic susceptibility within IMH regions, which corresponded closely with hypointense areas observed on T_2_/T_2_* images. Furthermore, in animal models, magnetic susceptibility values measured by QSM showed a strong correlation with tissue iron content determined by ex vivo mass spectrometry (R^2^ > 0.98), further validating its accuracy in iron quantification [16].

### 2.2. Lipid Peroxidation

Ferroptosis is characterized by iron-dependent lipid peroxidation, a process modulated by exosomal activity. Extracellular Vesicles (EVs) disrupt lipid homeostasis by delivering microRNAs and functional proteins that suppress the expression of glutathione peroxidase 4 (GPX4) and activate acyl-CoA synthetase long-chain family member 4 (ACSL4). This shift in enzyme activity redirects lipid metabolism toward the synthesis of pro-ferroptotic phosphatidylethanolamine hydroperoxides, thereby amplifying ferroptotic signaling and exacerbating oxidative stress. In obesity-related myocardial injury and atrial fibrillation, EVs have been shown to promote cardiac dysfunction by enhancing ferroptosis [17].

A central regulator of ferroptosis is glutathione peroxidase 4 (GPX4), a selenoprotein that utilizes glutathione (GSH) to reduce lipid hydroperoxides into nontoxic alcohols, thereby preventing peroxidation cascade. GPX4 activity depends on cellular GSH availability, which is maintained by the system Xc^−^ cystine/glutamate antiporter—a heterodimer of SLC7A11 and SLC3A2 [18]. Upon cystine uptake, intracellular reduction to cysteine supports GSH synthesis. Downregulation of system Xc^−^ impairs GSH production, inactivating GPX4 and leading to toxic lipid hydroperoxide (L-OOH) accumulation. In the presence of Fe^2+^, L-OOHs generate lipid radicals that damage cellular macromolecules and drive ferroptosis. Post-myocardial infarction (AMI), mitochondrial iron and lipid peroxides increase, while GPX4 expression is suppressed in early and mid-stage AMI. EVs can modulate GPX4 activity by interfering with system Xc^−^ function and GSH synthesis [19].

Lipid peroxidation is further amplified by the enzymatic remodeling of membrane phospholipids. Acyl-CoA synthetase long-chain family member (ACSL4) esterifies free polyunsaturated fatty acids (PUFAs), which are incorporated into membrane phosphatidylethanolamines (PEs) by Lysophosphatidylcholine Acyltransferase 3 (LPCAT3). Peroxidation of PUFA-PE releases toxic aldehydes such as 4-Hydroxynonenal (4-HNE) and Malondialdehyde (MDA), causing membrane damage and amplifying ferroptotic injury [20].

During myocardial ischemia–reperfusion (IR) injury, cardiac-derived extracellular vesicles (IR-EVs) exacerbate cardiomyocyte ferroptosis by delivering miR-155-5p, which suppresses the Nfe2l2 antioxidant pathway [21]. This process is accompanied by significant lipid peroxidation, characterized by the accumulation of toxic aldehyde species such as 4-hydroxynonenal (4-HNE). These findings suggest that 4-HNE not only acts as a key executor of ferroptosis but may also serve as a potential molecular imaging target.

### 2.3. The Central Regulatory Role of Nrf2

Nuclear factor erythroid 2-related factor 2 (Nrf2) serves as a master transcriptional regulator of cellular redox homeostasis, coordinating defense mechanisms against oxidative stress and lipid peroxidation. Under basal conditions, Kelch-like ECH-associated protein 1 (Keap1) targets Nrf2 for ubiquitin–proteasome degradation. Under oxidative stress, Nrf2 activation induces Sequestosome 1 (SQSTM1/p62) expression, leading to Keap1 degradation via selective autophagy and thereby relieving Nrf2 suppression. This positive feedback loop is reinforced by Nrf2-mediated transcription of autophagy-related genes (e.g., p62, Autophagy-related 5 (ATG5), and Unc-51 like autophagy activating kinase 1 (ULK1)), facilitating clearance of damaged cellular components [22]. As a central node in ferroptosis regulation, Nrf2 transcriptionally modulates genes involved in iron metabolism, glutathione biosynthesis, GPX4 expression, and lipid antioxidant defense. Nrf2 collaborates with heme oxygenase-1 (HO-1) to counter iron dysregulation, defective autophagy, and apoptosis in cardiovascular disease. Studies by Lv et al. demonstrate that Nrf2 activation ameliorates iron metabolism disorders in myocardial ischemia–reperfusion injury, highlighting its role as a molecular bridge connecting autophagy and iron homeostasis with therapeutic potential [23].

While this provides a clear biological target for imaging probe design, most current imaging techniques in preclinical studies remain limited to tracing the “location” and “distribution” of EVs, failing to effectively reflect the “status” of core ferroptosis events induced by EVs in target tissues. This limitation hinders the precise evaluation of EV-based therapeutic strategies. Integrating in vivo EV tracing with the visualization of key molecular events in ferroptosis therefore emerges as a central challenge that the subsequent sections of this review aim to address.

## 3. The Effect of Regulating Ferroptosis on Myocardial Infarction

Ferroptosis-related biomarkers show considerable promise for the diagnosis and therapeutic monitoring of AMI [24]. Key regulatory proteins—including mitochondrial ferritin (FtMt), glutathione peroxidase 4 (GPX4), system Xc^−^, and acyl-CoA synthetase long-chain family member 4 (ACSL4)—play central roles in modulating ferroptosis post-AMI [25]. FtMt confers protection by sequestering labile iron and attenuating Fenton chemistry. GPX4 and system Xc^−^ (a heterodimer of SLC7A11 and SLC3A2) help maintain redox balance via glutathione-dependent lipid peroxide reduction, whereas ACSL4 facilitates ferroptosis by driving polyunsaturated fatty acid incorporation into membrane phospholipids, thereby promoting peroxidation [26,27] (Table 1).

Clinically, AMI patients exhibit altered iron metabolism profiles, including decreased serum iron levels, increased total iron-binding capacity (TIBC), and elevated serum ferritin (SF). Among these, serum iron has been identified as an independent cardioprotective factor, with lower levels associated with larger infarct size and worse outcomes. However, circulating iron levels exhibit marked biological variability (diurnal rhythm, inflammation, and hemodilution), limiting their reliability as a stand-alone biomarker in the acute setting [37].

Targeting ferroptosis has thus emerged as a promising therapeutic strategy for AMI [38]. Current interventions primarily operate through three mechanisms: (1) ferroptosis inhibitors (e.g., ferrostatin-1 and liproxstatin-1) that scavenge lipid radicals and block peroxidation propagation; (2) antioxidants such as N-acetylcysteine that support GPX4 activity by replenishing glutathione; and (3) iron chelators like deferoxamine that sequester redox-active iron [39]. Preclinically, ferrostatin-1 attenuates cardiomyocyte ferroptosis, reduces infarct size, and improves cardiac function by suppressing myocardial enzyme release [40,41]. Similarly, deferoxamine limits ischemia–reperfusion injury by forming inert iron complexes, thus preventing iron-driven lipid peroxidation and myocardial damage [42,43].

## 4. Mechanisms of Extracellular Vesicles in Regulating Ferroptosis

EVs function as key mediators of intercellular communication by packaging and transferring bioactive molecules—including proteins, lipids, and nucleic acids—between cells. They contribute to both physiological homeostasis and pathological processes, and their natural biocompatibility and targeting specificity make them attractive nanocarriers for therapeutic delivery [44]. Emerging evidence highlights a complex bidirectional regulatory relationship between EVs and ferroptosis, wherein EVs can either promote or inhibit ferroptosis depending on their cellular origin and cargo composition.

### 4.1. EVs as Promoters of Ferroptosis

Under pathological conditions, certain EVs can propagate ferroptotic signals. For instance, adipose tissue macrophage-derived exosomes deliver miRNAs that suppress SLC7A11 expression, thereby inhibiting glutathione synthesis and promoting ferroptosis in obesity-induced cardiac injury [45]. Similarly, cardiac fibroblast-derived exosomes transport miR-23a-3p, which targets SLC7A11, to cardiomyocytes, thereby exacerbating ferroptosis in atrial fibrillation [46]. These findings demonstrate how EVs can serve as vehicles for transferring pro-ferroptotic molecules between different cell types in the cardiovascular system. Zhao [47] discovered that in obese conditions, EVs from adipose tissue macrophages carry miR-140-5p, which promotes ferroptosis in cardiomyocytes by targeting SLC7A11, inhibiting GSH synthesis and worsening myocardial injury. Myocardial ischemia–reperfusion (IR) injury itself stimulates the release of a class of EVs from the heart that possess “pro-inflammatory” and “pro-death” properties. Animal studies have confirmed that transferring EVs isolated from IR-injured hearts (IR-EVs) to healthy hearts or cardiomyocytes is sufficient to directly induce or exacerbate ferroptosis [25]. The core mechanism involves the significant enrichment of miR-155-5p within IR-EVs. Upon delivery into recipient cardiomyocytes, this miRNA directly targets and binds to the 3′UTR region of Nfe2l2 (also known as Nrf2), inhibiting its translation and expression. The subsequent downregulation of Nrf2 inactivates the downstream antioxidant response element (ARE) pathway, leading to decreased expression of antioxidant enzymes such as HO-1, Fth1, and SLC7A11 [48]. This ultimately weakens the defense capacity of cardiomyocytes against lipid peroxidation attacks, increasing their susceptibility to ferroptosis. This discovery reveals a crucial “EVs-miRNA-antioxidant defense” axis in IR injury, offering new therapeutic targets for intervention.

In patients with AMI, abundant exosomes are present within coronary thrombi. These thrombus-derived exosomes (TEs) can induce significant ferritinophagy in cardiomyocytes, characterized by increased autophagosomes, elevated reactive oxygen species (ROS) production, iron overload, and apoptosis—an effect reversible by the ferroptosis-specific inhibitor Fer-1 [49]. Mechanistically, TEs deliver the long non-coding RNA FENDRR, which synergizes with P53 to upregulate the expression of m6A modification-related proteins (YTHDF1/YTHDF3) in cardiomyocytes. This activates NCOA4-mediated ferritinophagy, leading to ferritin degradation, the release of labile iron, and the subsequent triggering of ferroptosis. This is the first study to directly link the pathological entity of “thrombus” to “myocardial ferroptosis” via EVs, providing a novel perspective for understanding remote organ damage and post-reperfusion complications after myocardial infarction [50].

### 4.2. EVs as Inhibitors of Ferroptosis

Conversely, several studies have revealed the protective role of EVs against ferroptosis. Mesenchymal stem cell (MSC)-derived exosomes have been shown to alleviate myocardial injury by transferring miR-26b-5p, which targets SLC7A11 to inhibit ferroptosis in AMI [51].

Research has revealed that exosomes derived from bone marrow mesenchymal stem cells (BMSCs) overexpressing the transcription factor GATA-4 can enrich miR-330-3p and deliver it to cardiomyocytes, where it targets and inhibits the expression of the deubiquitinating enzyme BAP1, thereby upregulating SLC7A11, a key subunit of the cystine/glutamate antiporter system [25]. The activation of this axis not only effectively inhibits hypoxia/reoxygenation (H/R)-induced cardiomyocyte ferroptosis but also reduces the opening of the mitochondrial permeability transition pore (mPTP), preserving mitochondrial functional integrity [52]. Notably, this process is also accompanied by an increase in hydrogen sulfide (H_2_S) content and regulation of the Keap1/Nrf2 signaling pathway, suggesting that GATA-4-EVs exert synergistic antioxidant and anti-ferroptotic effects through multiple targets [25].

In a murine model of viral myocarditis induced by Coxsackievirus B3 (CVB3), human umbilical cord mesenchymal stem cell-derived EVs (hucMSC-EVs) were shown to deliver let-7a-5p, which targets SMAD2, subsequently promoting ZFP36 expression [53]. This mechanism significantly inhibited cardiomyocyte ferroptosis and improved cardiac function. These findings broaden the potential application of anti-ferroptotic EVs in non-ischemic cardiomyopathies.

EVs derived from human umbilical cord MSCs play a protective role in treating AMI by carrying miR-23a-3p, which inhibits ferroptosis in cardiomyocytes and reduces myocardial injury [54,55]. In addition, EVs from pericardial adipose tissue regulate iron metabolism through Adipsin, targeting IRP to maintain iron homeostasis in cardiomyocytes and inhibit ferroptosis [56].

Similarly, human umbilical cord blood-derived MSC exosomes attenuate myocardial injury by suppressing ferroptosis through the upregulation of GPX4 in AMI mice [57]. Bone marrow MSC-derived exosomal lncRNA Mir9-3hg has been found to suppress cardiomyocyte ferroptosis in ischemia–reperfusion mice via the Pum2/PRDX6 axis [58].

Research has demonstrated that macrophage-derived EVs inherit the transferrin receptor (TfR) from their parent cells, enabling them to function like a “biological sponge” that binds and clears excess free iron ions in myocardial infarct regions. This direct mechanism of action, based on functional membrane proteins, is fundamentally distinct from traditional miRNA regulatory paradigms, offering both high efficiency and specificity [59]. In animal models of myocardial infarction, local injection of macrophage-derived EVs significantly attenuated oxidative stress induced by iron overload, inhibited ferroptosis, and improved cardiac function [60].

Furthermore, EVs derived from human induced pluripotent stem cells (iPSCs) overexpressing OTUD5 can deliver OTUD5 protein into recipient endothelial cells, where they regulate the NF-κB/p65 signaling pathway and restore GPX4 expression, thereby effectively inhibiting endothelial cell ferroptosis [61]. Importantly, treatment with these EVs promoted angiogenesis, improved myocardial perfusion, and enhanced cardiac function following myocardial infarction [62]. This line of investigation suggests that the protective effects of anti-ferroptotic EVs are not limited to cardiomyocytes but also extend to endothelial cells that constitute the microvascular environment, offering a new paradigm for holistic cardiac repair after myocardial infarction.

Furthermore, exosomes derived from pericardial adipose tissues attenuate cardiac remodeling following AMI by delivering Adipsin to cardiomyocytes, where it modulates iron homeostasis via hepcidin suppression, thereby increasing iron export through ferroportin and reducing labile iron pool-mediated oxidative stress [63]. The dual role of EVs in ferroptosis regulation presents significant therapeutic opportunities. Engineered EVs can be designed to deliver ferroptosis inhibitors specifically to damaged myocardium. For example, EVs loaded with ferroptosis-suppressing miRNAs or proteins such as GPX4 offer a targeted approach to ameliorate myocardial injury [64]. Additionally, the ability of EVs to cross biological barriers makes them ideal for delivering therapeutic cargo to sites of injury that are otherwise challenging to access. Pro-ferroptotic EVs exhibit the following common characteristics: (1) they are predominantly derived from cells under stress or injury conditions (such as ischemic myocardium or thrombotic cells); (2) they are enriched with pro-oxidant and pro-death molecules (e.g., miR-155-5p, lncRNA FENDRR); (3) their mechanisms of action converge on suppressing antioxidant defense systems (such as the Nrf2 pathway) or activating iron-dependent cell death pathways (e.g., ferritinophagy) [65]; and (4) their pathogenic effects are more pronounced when recipient cells are under stress, suggesting a synergistic interaction between “damage signals” and the “susceptible microenvironment”.

## 5. Generation, Isolation, and Functional Engineering Strategies of Extracellular Vesicles

The research on and application of extracellular vesicles as a natural endogenous delivery system highly depend on efficient isolation techniques, precise in vivo tracking methods, and engineering modifications designed to enhance their targeting and therapeutic efficacy.

### 5.1. Biogenesis and Isolation

**Figure 2:**
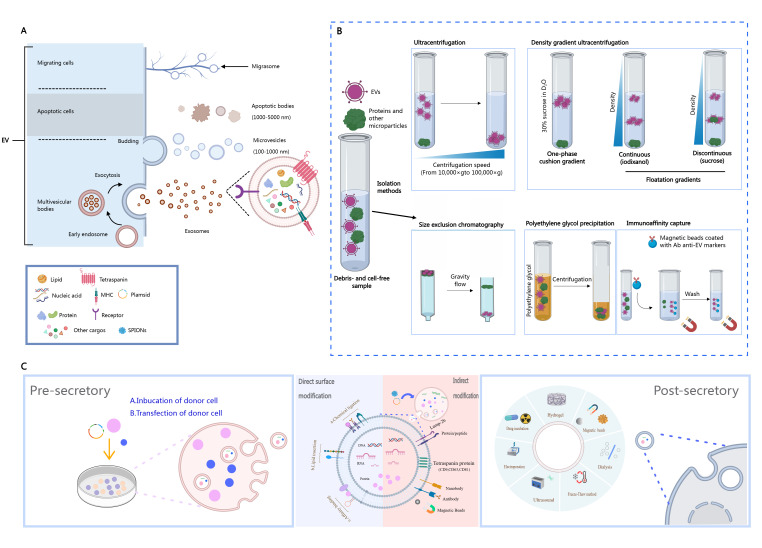
(**A**) Schematic overview of EV biogenesis: exosomes (endosomal pathway), apoptotic bodies (programmed cell death), and migratory bodies (retraction fibers), all membrane-bound with cargo transport functions. (**B**) Isolation methods: ultracentrifugation (size/density), density gradient (sucrose/iodixanol separation), size-exclusion chromatography (pore retention), PEG precipitation (aggregation), and immunoaffinity capture (antibody-specific binding). (**C**) Classification of EV cargo loading methods (pre-/post-secretory) and core engineering strategies: direct surface modification versus donor cell modification via EV biogenesis pathway engineering.

The biogenesis of extracellular vesicles begins with the endosomal system of the cell (Figure 2A). Vesicles budded from the endoplasmic reticulum fuse with the Golgi apparatus to form early endosomes. During endosomal maturation, coordinated protein sorting drives the inward budding of the limiting membrane, generating intraluminal vesicles within multivesicular bodies (MVBs). Finally, MVBs fuse with the plasma membrane, releasing these vesicles into the extracellular space as EVs [66]. EVs are characterized by a bilayer lipid membrane, which confers high biocompatibility and stability, and enables the encapsulation of diverse biomolecular cargo (such as proteins, nucleic acids, and small molecules), thereby facilitating complex intercellular communication.

The efficient isolation of EVs from complex biological samples is fundamental for functional studies and applications. Commonly used methods include the following (Figure 2B):

Ultracentrifugation: The gold standard method for separation based on EV size and density.

Density gradient centrifugation: This process enables finer separation using media such as sucrose or iodixanol. Size-exclusion chromatography: This process separates EVs by size using porous matrices, effectively preserving their biological activity.

PEG precipitation: This approach induces EV aggregation via volume exclusion-driven dehydration, not polymerization. While simple and convenient, this method can compromise EV integrity, mask surface epitopes, and co-precipitate contaminants, limiting its use for functional studies. Immunoaffinity capture: This method employs antibodies against EV surface markers (e.g., CD63, CD81) to achieve high-purity isolation without altering the EV structure or function.

### 5.2. Labeling Strategies and Imaging Probe Loading

The labeling of EVs is essential for their clinical identification, in vivo tracking, and functional studies. Strategies for loading imaging probes or therapeutic agents are broadly categorized into direct and indirect methods (Figure 2C).

#### 5.2.1. Direct Labeling

Direct labeling involves introducing markers into EVs after their secretion through physical or chemical approaches. Physical methods: Techniques such as freeze–thaw cycles, sonication, dialysis, and electroporation utilize concentration gradients or transient membrane disruption to passively load therapeutic agents or contrast agents (e.g., superparamagnetic iron oxide nanoparticles, SPIONs) into EVs. For instance, electroporation of EVs resuspended with SPIONs enables efficient encapsulation, allowing non-invasive tracking via magnetic resonance imaging [67]. Chemical methods: Functionalization is achieved by affinity-based conjugation or covalent bonding of peptides, antibodies, or aptamers onto the EV surface through simple incubation, supporting targeted applications.

#### 5.2.2. Indirect Labeling

Indirect labeling involves engineering parental cells to secrete EVs that inherently carry the desired markers. Parental cell labeling: Incubation of parental cells with labeling agents (e.g., ultrasmall superparamagnetic iron oxide particles, USPIO) allows for the incorporation of these markers into EVs during biogenesis, resulting in labeled EVs upon secretion. Genetic engineering: Transfection of parental cells with plasmid or viral vectors encoding fluorescent proteins (e.g., GFP), luciferases (e.g., Fluc), or fusion proteins of EV membrane-anchored scaffolds and target genes enables the secretion of EVs carrying the corresponding reporter genes or functional proteins [68].

### 5.3. Functional Engineering Strategies

To enhance the targeting specificity, drug-loading efficiency, or therapeutic efficacy of EVs, various engineering strategies have been developed (Figure 2C). Membrane fusion: Hybrid vesicles are formed by fusing cationic liposomes with EV membranes via electrostatic interactions or freeze–thaw cycles. This strategy significantly enhances EV-receptor binding affinity and cellular uptake efficiency. Compared to free drugs or conventional liposomal carriers, membrane-fused hybrid EVs demonstrate a 3–4-fold improvement in chemotherapeutic drug delivery efficiency [69]. Covalent modification: Stable ligand conjugation is achieved through covalent bonds between functional groups on the EV surface (e.g., amines, carboxyls, and thiols) and targeting molecules. This approach enables precise surface functionalization while preserving EV structural integrity and bioactivity [70]. Non-covalent modification: This strategy facilitates the encapsulation of therapeutic cargo (e.g., CRISPR-Cas9 plasmids) or anchoring of surface ligands through weaker interactions such as hydrophobic effects or electrostatic adsorption. A representative example employs click chemistry (a bioorthogonal reaction that enables covalent conjugation under mild conditions) to conjugate neuraminidase-1-targeting peptides onto EVs, achieving dual optimization: (1) enhanced glioma-specific targeting, and (2) improved blood–brain barrier penetration via surface property modulation [71]. Metabolic labeling: Azide-modified mannose or amino acids are supplemented into the culture medium of parental cells. These substrates are metabolically incorporated into secreted EVs. Subsequent copper-catalyzed azide-alkyne cycloaddition (CuAAC) enables covalent conjugation of alkyne-bearing fluorescent probes or therapeutic payloads to EVs. This method achieves >90% labeling efficiency while maintaining EV structural integrity and bioactivity, making it suitable for real-time in vivo tracking (Figure 2A,C) [72].

After cardiac bypass grafting (CPB) surgery, the EV SEMA5A interacts with miR-143-3p. This interaction prevents miR-143-3p from binding to target mRNAs like BCL2 and SLC7A11, which protects cardiomyocytes from ischemia/reperfusion (I/R) injury [73].

Myocardial ischemia–reperfusion injury raises inflammation levels in the heart and also triggers systemic inflammatory responses via blood circulation. MiR-155-5p in IR EVs can promote M1 polarization of macrophages by activating the JAK2/STAT1 signaling pathway [74]. Treatment with GW4869 can reduce the release of IR EVs in circulation, decrease the levels of pro-inflammatory cytokines (such as IL-1β and IL-6) and chemokines (such as CXCL1 and CXCL2) in circulation, and decrease the expression of pro-inflammatory genes in distant organs [75]. The pericardial drainage pathway enhances immune activation in the mediastinal lymph nodes following AMI [76]. EVs injected into the pericardial cavity accumulate in mediastinal lymph nodes and induce the differentiation of regulatory T cells (Tregs), promoting cardiac repair. Exosomes are taken up by MHC-II+ antigen-presenting cells (APCs) and activate FOXO3 through the PP2A/p-Akt/FOXO3 pathway. FOXO3 dominates the expression of cytokines in APCs, the differentiation of Tregs, and their increased distribution in the heart, contributing to the resolution of cardiac inflammation and cardiac repair [77,78].

## 6. Application of Molecular Imaging in EVs

The efficiency of EV homing is governed by multiple factors, including the complex microenvironment of the infarcted heart during repair and dynamic changes in vascular permeability within ischemia–reperfusion-affected regions. In the myocardium, the initiation and execution of ferroptosis are confined to a narrow temporal window, with oxidative stress typically peaking within hours after reperfusion. The therapeutic alignment between EV-mediated regulation and ferroptosis dynamics therefore depends critically on the retention half-life of EVs within target tissues.

Molecular imaging refers to a category of medical imaging techniques designed to visualize, characterize, and quantify biological processes at the molecular and cellular levels. In contrast to conventional anatomical imaging, it focuses on early disease detection and precise pathological profiling. This approach has demonstrated significant clinical value in AMI research, enabling the in situ visualization of metabolic activity and molecular alterations. By employing targeted molecular probes, molecular imaging can monitor key molecular events throughout the progression of AMI, thereby facilitating early diagnosis and guiding treatment strategies [79]. Several molecular imaging modalities—including positron emission tomography (PET), single-photon emission computed tomography (SPECT), magnetic resonance imaging (MRI), and optical imaging—are now routinely utilized in AMI detection (Table 2).

### 6.1. Fluorescence Imaging

Fluorescence imaging enables the real-time tracking of extracellular vesicle (EV) distribution and dynamics through optical labelling, offering the direct visualization of EV targeting and mechanisms in myocardial infarction. Fluorescent labelling employs two primary strategies, namely direct labelling using lipophilic dyes (e.g., PKH67, PKH26, DiR, DiD, and DiI) or quantum dots that integrate into EV membranes [80], and indirect labelling via genetic engineering of parent cells to express fluorescent proteins (e.g., GFP, and tdTomato), enabling sensitive and sustained EV tracking in specific cellular contexts [81]. While widely applied in cellular studies to monitor EV uptake, release, and intercellular transfer, fluorescence imaging is constrained in deep-tissue applications by limited signal penetration [82].

**Figure 3:**
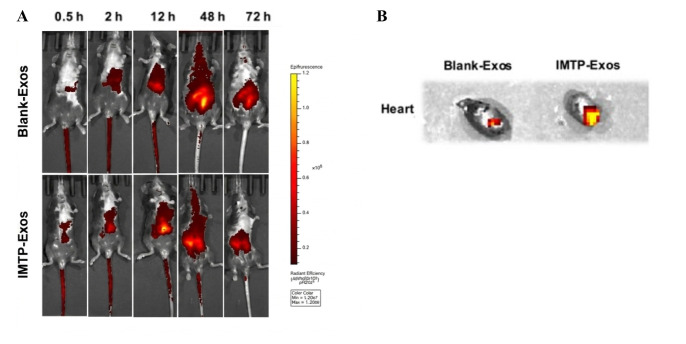
(**A**) Mice underwent AMI surgery and received DiR-labeled blank-Exos or IMTP-Exos treatment through tail intravenous injection. Fluorescence signals were detected up to 72 h after injection. (**B**) The intensity of fluorescence signals from the ischemic myocardium was quantified using an IVIS. Figures were adapted with permission from Li et al. [84].

Li et al. [83] delivered PKH26-labelled AR Neo EVs via alginate microspheres, showing prolonged cardiac retention over 14 days. Another study using DIR-labelled tanshinone IIA-preconditioned MSC exosomes confirmed myocardial retention for 72 h. Chen et al. further demonstrated that cardiac targeting peptide (CTP)-modified EVs exhibited enhanced cardiac accumulation and promoted myocardial repair by reducing oxidative stress, while unmodified exosomes predominantly accumulated in the liver, spleen, and lungs, promoting myocardial repair and reducing ischemic area (Figure 3).

### 6.2. BLI

BLI utilizes luminescent reactions in living systems for the high-sensitivity, real-time tracking of EVs. Direct labelling involves conjugating luciferases—such as firefly (Fluc), *Renilla* (Rluc), or Gaussian luciferase (Gluc)—to EVs, generating light upon substrate addition. Indirect labelling employs genetic engineering of parent cells to produce luciferase-carrying EVs, enabling non-invasive in vivo imaging upon substrate administration [85]. BLI offers high sensitivity, low background, making it suitable for longitudinal EV biodistribution studies, though its use is restricted by limited penetration depth and the need for exogenous substrates [86,87].

**Figure 4:**
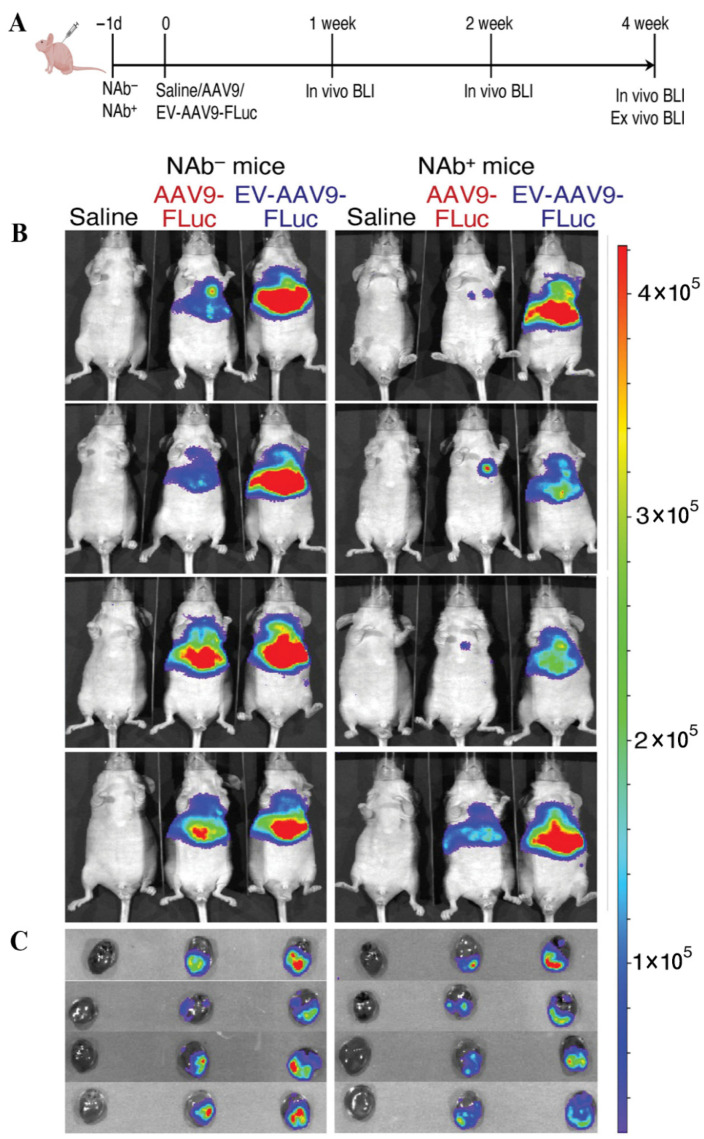
EV-AAVs deliver genes more efficiently than AAVs in the presence of NAb in vivo. (**A**) Study design. (**B**) Bioluminescent images of nude mice at 4 weeks after injection. (**C**) Ex vivo imaging of hearts. Figures adapted with permission from Li et al. [90].

BLI has proven valuable in evaluating cardiac-targeted EV therapies. For instance, EV-AAV9 vectors carrying luciferase fused to EV membrane protein CD63 enabled real-time in vivo tracking. BLI confirmed that EV-AAV9-SERCA2a restored ejection fraction and fractional shortening in AMI models even in the presence of neutralizing antibodies, whereas conventional AAV was ineffective [88] (Figure 4). In AMI models, BLI dynamically visualizes EV homing to infarcted myocardium, permitting real-time analysis of how EVs modulate the local microenvironment. Its high sensitivity and low background are particularly useful for investigating ferroptosis and other molecular processes underlying cardiac injury and repair [89].

### 6.3. MRI

MRI is a three-dimensional imaging modality based on nuclear magnetic polarization, valued for its high soft-tissue resolution and absence of ionizing radiation [91]. Superparamagnetic iron oxide nanoparticles (SPIONs), widely used as clinical contrast agents for over two decades, effectively shorten proton relaxation times and enhance MRI contrast due to their superparamagnetism, nanoscale dimensions, and biocompatibility.

EVs can be labeled for MRI via direct or indirect strategies. Direct labeling incorporates magnetic nanoparticles (e.g., SPIO and, USPIO) into EVs using techniques such as electroporation. Indirect labeling involves engineering parental cells to express magnetic proteins or those that bind magnetic nanoparticles. An innovative approach loads magnetic nanoparticles (MNPs) into exosomes, leveraging their native membrane structure to shield MNPs from biological interference while improving vascular penetration. This strategy can enhance MRI contrast by 2–3-fold and, when combined with external magnetic navigation, enables targeted delivery of therapeutic exosomes with real-time tracking, forming a theranostic platform [92].

However, its relatively low sensitivity often necessitates high EV concentrations for a detectable signal, and it faces limitations including long scan times and incompatibility with certain metallic implants [93]. To overcome sensitivity constraints, SPIO/USPIO labeling remains commonly employed.

**Figure 5:**
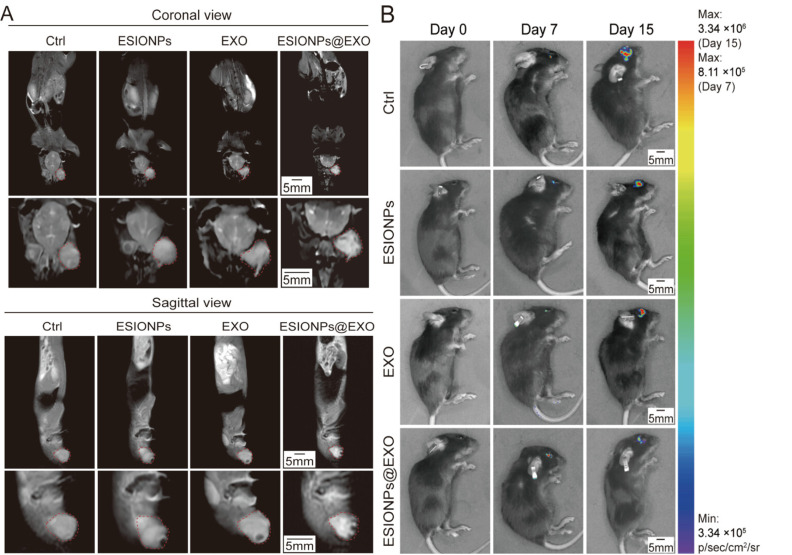
(**A**) ESIONPs@EXO enhance coronal (**up**) and sagittal (**down**) T1-weighted MRI of tumor-bearing mice in vivo. Red dotted line indicates tumors. (**B**) Bioluminescence images. Figures adapted with permission from Zhang et al. [95].

Studies confirm that magnetically labeled exosomes serve as effective and stable MRI contrast agents. For example, macrophages incubated with ultra-small iron oxide nanoparticles (ESIONPs) produced engineered exosomes (ESIONPs@EXO) that not only enhanced MRI contrast but also suppressed pathological angiogenesis, induced ferroptosis, and exerted immunotherapeutic effects, demonstrating a robust theranostic potential [94] (Figure 5). Lee et al. [95] showed that USPIO-labeled mesenchymal stem cell-derived nanovesicles (NVs) enabled high-field (9.4T) MRI of infarcted rat myocardium. Magnetic navigation doubled NV retention in the infarct zone and significantly improved cardiac function.

In therapeutic applications, doxorubicin-loaded SPION-EVs enhance tumor suppression under magnetic guidance [96]. Ethylene glycol-modified iron oxide nanocubes (MFRNs) exhibit improved biocompatibility and stability; when administered intravenously in acute myocardial infarction (AMI) models and targeted using a 0.4 T magnet, they accumulate precisely in infarcted myocardium, as confirmed by T2*-weighted MRI at 7 T [97]. Increasing SPION-exosome concentrations enhances negative contrast on T2*-weighted images, enabling quantitative tracking [98].

Transferrin-conjugated Fe_3_O_4_ nanoparticles (M-Tf) facilitate the production of engineered exosomes (D-Exos/miR21i-L17E) via magnetic separation and electrostatic interaction, ensuring high stability and efficient co-loading of doxorubicin and chol-miR21i. L17E peptide modification further improves RNA transfection efficiency [92]. Liu et al. [99] developed antibody-modified core–shell magnetic nanoparticles (GMNPs) that capture endogenous CD63-positive EVs and guide them to infarcted myocardium, improving cardiac function and ejection fraction. Zheng et al. [100] encapsulated SPIO-His in EVs via electroporation and used T2*-MRI with R2 mapping to quantify SPION concentration and distribution for regenerative medicine.

Beyond SPIONs, FTH1 serves as a natural MRI contrast agent when expressed in mesenchymal stem cell-derived EVs via lactadherin fusion, enabling EV visualization [101]. Quantitative susceptibility mapping (QSM) detects iron changes in reperfused AMI, with magnetic susceptibility correlating with tissue iron content, coronary occlusion duration, and clinical features such as microvascular obstruction [102].

Magnetic particle imaging (MPI) offers high sensitivity (nM-level SPION detection) and spatial resolution (∼100 μm), with no signal depth attenuation, enabling quantitative 3D iron tracking over months [103,104]. However, inter-system signal variability exceeding 3-fold necessitates standardization for cross-institutional reproducibility. Alternatively, Sancho-Albero et al. [105] developed fluorinated EVs incorporating a PERFECTA probe (^39^F-MRI), demonstrating tumor-targeting capability and systemic biodistribution visualization after intravenous injection.

### 6.4. SPECT and PET

Nuclear imaging—including single-photon emission computed tomography (SPECT) and positron emission tomography (PET)—visualizes metabolic activity and physiological function using radioactive tracers. It is often combined with CT or MRI to provide integrated anatomical and functional information.

The radiolabeling of EVs is achieved through two main strategies: surface labeling and intracavity labeling. Surface labeling involves genetic engineering, direct incorporation of radionuclides into membrane components, or the use of chelators to conjugate isotopes (e.g., ^99^mTc, ^111^In, ^18^F, ^64^Cu, and ^68^Ga) to the EV surface via chemical or physical adsorption [106]. Intracavity labeling employs remote loading or ionophore-chelator systems to encapsulate radioactive metal complexes within the vesicle lumen. A key challenge in EVs radiolabeling is preserving vesicle integrity, as modifications may alter biodistribution or function and limit diagnostic or therapeutic utility. The precise localization of radionuclides within EVs also remains uncertain, complicating image interpretation [107,108].

Yang et al. [109] used ^99m^Tc-labeled MSC-EVs in SPECT imaging, showing accumulation in the liver and bladder within 6 h, indicating systemic clearance, while demonstrating the ability to cross vascular barriers and target damaged brain regions by 24 h. These EVs carried miR-214-3p, which suppressed ferroptosis via GPX4 upregulation and ACSL4 downregulation. Shi et al. [110] engineered M2 macrophage-derived EVs with polydopamine (PDA) coating, conjugated to brain-targeting peptide RVG29 and multimodal probes (SPIO, ^125^I, fluorophores). In the field of oncology research, strategies integrating enhanced cellular uptake based on the tumor microenvironment’s pH with radiotherapy have been developed [111]. ^111^In is suitable for longer-term EV tracking due to its extended half-life and low gamma energy. ^64^Cu, in contrast, emits high-energy positrons that enable high-resolution PET imaging. ^64^Cu is typically conjugated to EV surfaces via chelators such as NODAGA. ^64^Cu-NODAGA-labeled EVs exhibit specific and time-dependent pulmonary accumulation with high radiostability [112]. It is commonly linked to EV surface groups using chelators like DOTA or NOTA [113]. One strategy is deferoxamine (DFO)-assisted labeling of small EVs with [^89^Zr]Zr(oxinate)_4_ [114], this approach demonstrates effective tissue uptake of EVs, reveals their natural targeting specificity, and highlights significant differences in biodistribution across organs and between sexes. These findings provide a research direction for targeted therapy of myocardial infarction [115]. Myocardial perfusion imaging is a commonly used non-invasive tool for assessing the severity of coronary artery disease. Studies indicate that myocardial perfusion and functional parameters correlate with the levels of circulating EVs in patients, reflecting dynamic changes in EVs during disease progression.

SPECT and PET imaging provide high sensitivity and quantitative capability in EV research, enabling detailed analysis of their biodistribution and metabolic behavior and early detection of myocardial ischemia and infarction. However, these modalities involve ionizing radiation, high operational costs, and require advanced instrumentation and expertise. Their use is further constrained by the physical half-life of radionuclides.

In vivo imaging of EVs provides critical insights into their biodistribution, targeting efficiency, and biological roles in intact physiological systems. This approach enables real-time monitoring of EV biogenesis, secretion, uptake, and intercellular communication, offering a dynamic perspective on their functional cargo delivery. Although murine models offer relevant physiological parallels to humans, practical constraints—including limited imaging resolution for small EVs, anatomical complexity that impedes optical clarity, and discrepancies between EV accumulation sites and functional activity zones—often lead researchers to employ simpler model organisms. A summary of current in vivo imaging strategies and molecular markers used to investigate EV biology across these models is presented in Table 3.

### 6.5. CT Imaging

CT plays a crucial role in disease diagnosis by measuring the absorption of X-rays in the body, providing images with high temporal and spatial resolution. However, due to the limited contrast of CT imaging, difficulties may be encountered when using CT to track EVs. Recent studies have highlighted the potential of gold nanoparticles labeled EV from stem cells in revealing the migration and homing patterns of EV in cardiovascular diseases [130].

Gong’s team [128] has developed a novel targeted delivery system that combines glucose-modified gold nanoparticles (GNPs) with EVs derived from MSCs to construct GNP EVs complexes. This complex exhibits significant advantages in a mouse model of focal AMI, such as precise targeting, efficient enrichment, and enhanced targeting through glucose modification. Within 24 h after intranasal administration, CT imaging shows specific accumulation of GNPs EVs in the AMI area, without causing extracellular vesicle aggregation, ensuring its structural stability and biological activity. Dynamic imaging monitoring was achieved using in vivo CT technology, which successfully tracked the spatiotemporal distribution of labeled EVs in AMI mouse models at 4 and 24 h after injection, confirming their rapid lesion homing ability. This complex offers a dual-functional platform that enhances cardiac repair after AMI. It achieves this through targeted delivery of therapeutic components, integrating real-time imaging guidance with therapeutic intervention capabilities. This approach is expected to advance the precise development of cardiac regenerative medicine [131].

### 6.6. Artificial Intelligence

With the widespread adoption of artificial intelligence, machine learning—especially deep learning—has progressed rapidly. Deep learning enables automatic feature extraction from large-scale datasets, reducing dependence on manual feature engineering and substantially improving the intelligence of image analysis and interpretation. Cardiovascular disease data are notably rich and heterogeneous. In a study on coronary artery disease classification based on SPECT myocardial perfusion images, 630 patient cases were allocated, with 85% used for training and 15% for testing. From the training set, an additional 15% was held out as a validation set for hyperparameter tuning and early stopping [132]. For complex models involving multiple subtasks, a stratified splitting strategy can be adopted. In a study on right ventricular myocardial infarction segmentation, a multicenter dataset comprising 213 CMR images was divided into training and test sets at a 4:1 ratio, and five-fold cross-validation was implemented within the training set [133]. During cross-validation, data preprocessing steps—such as feature normalization—must be strictly fitted on each training fold before being applied to the corresponding validation fold. As emphasized in a machine learning tutorial, feature scaling parameters (e.g., the mean and standard deviation for Standard Scaler) should be computed solely on the training fold and then applied to both the training and validation folds, thereby preventing information leakage from the validation set into the training process. This principle is particularly critical in myocardial infarction imaging, where image intensity values may exhibit systematic biases due to variations in imaging equipment or acquisition protocols. It is also important to note that cross-validation results alone cannot replace evaluation on an independent test set.

Beyond conventional cardiovascular imaging modalities, the application of AI to extracellular vesicle (EV)-based imaging presents both unique opportunities and distinct challenges. EV imaging datasets—generated from fluorescence, bioluminescence, PET, or MRI—are characterized by inherent heterogeneity arising from variations in EV size, surface marker expression, labeling efficiency, and isolation methods. Deep learning offers significant benefits in this context, including automated segmentation of EV signals from high-background noise, quantitative analysis of dynamic biodistribution patterns, and extraction of latent features that correlate with myocardial infarction severity or recovery. These capabilities reduce reliance on manual thresholding and enable more reproducible EV tracking. However, several limitations warrant consideration. First, EV signal intensity is susceptible to systematic biases from imaging equipment, acquisition protocols, and labeling efficiency, making rigorous cross-validation essential. Second, the limited size of EV imaging datasets—often constrained by experimental throughput—may hinder the training of complex deep learning models without overfitting. Third, batch effects introduced by differences in EV isolation or labeling across experiments can compromise model generalizability. Therefore, stratified splitting strategies that ensure that diverse EV populations are represented across training, validation, and test sets are particularly critical. As with conventional imaging, cross-validation alone cannot replace evaluation on an independent test set, and for EV studies, this is especially important given the potential for technical variability across multi-center or multi-batch experiments.

## 7. Discussion

The complexity of the regulatory network related to ferroptosis and associated EVs constitutes a major obstacle to the clinical translation of current theoretical frameworks. First, the spatiotemporal dynamics of ferroptosis in myocardial infarction remain poorly defined. The timing of initiation, peak activity, and cell-type-specific heterogeneity of ferroptosis in the infarct core, risk region, and remote myocardium have yet to be systematically characterized [134], leading to ambiguity in the selection of intervention timing and therapeutic targets. Second, the “loading-effect” relationship through which EVs regulate ferroptosis lacks systematic elucidation. Existing studies are largely confined to correlative analyses or functional validation of individual molecules, making it difficult to uncover the regulatory networks underlying the synergistic action of multiple molecules. To address these challenges, future research should integrate single-cell sequencing with spatial transcriptomics to construct a high-resolution “ferroptosis spatiotemporal atlas” in animal models of AMI [135]. This would enable the precise delineation of ferroptosis status across different pathological stages and microenvironments. In parallel, high-throughput functional screening platforms for EV cargo—such as those incorporating CRISPR library screening technologies—should be established to systematically dissect regulatory networks and identify combinations of cargo molecules with core therapeutic value. These efforts will provide both a theoretical foundation and technical support for the development of imaging probes and precision intervention strategies.

Translating ferroptosis from an in vitro concept to an in vivo visualizable target faces two major core challenges: probe specificity and interference from labeling methods. On one hand, currently available probes for ferroptosis imaging, such as QSM for detecting iron ions or fluorescent probes for detecting reactive oxygen species, only reflect the associated events or downstream effects of ferroptosis, rather than its core molecular events (e.g., GPX4 inactivation, specific accumulation of phospholipid peroxides). This leads to ambiguity in the interpretation of imaging results. To address this, there is an urgent need to promote the development of specific probes targeting core ferroptosis markers, such as PET tracers targeting inactivated GPX4 or fluorescent/chemiluminescent probes with high selectivity for phospholipid peroxides. Before specific probes become available, priority could be given to translating the relatively mature QSM technology for use as a surrogate indicator for assessing tissue iron deposition [136]. On the other hand, to track EVs, commonly used fluorescent dyes or protein labeling methods may alter the membrane physical properties, cargo composition, or cellular uptake efficiency of EVs, thereby interfering with their native functions. Therefore, the active development of labeling techniques that minimally interfere with EV function is essential, such as metabolic labeling or genetically encoded nanoprobes [137]. Furthermore, establishing “post-labeling functional validation standards” is crucial to ensure that labeled EVs show no significant differences in biological function compared to unlabeled EVs in in vitro cellular assays, thereby providing a reliable technical foundation for in vivo tracing.

Ferroptosis, as a pathway of programmed cell death, holds biological significance far beyond the passive “clearance” of damaged cells; it is deeply involved in tissue remodeling and immune modulation following injury. Consequently, non-selective inhibition may introduce a range of potential risks [138]. First, regarding the delay in damage clearance and immune remodeling, damage-associated molecular patterns released during ferroptosis serve as key signals initiating the inflammatory response after cardiac injury. An appropriate level of inflammation is essential for clearing dead cells and debris; premature or excessive inhibition of ferroptosis may lead to the retention of necrotic material, paradoxically worsening the microenvironment [139]. Second, concerning the interference with immune cell function, recent studies suggest that immune cells, such as macrophages, may also rely on ferroptosis for their normal functions. Non-specifically inhibiting ferroptosis in these immune cells could disrupt their phagocytic activity and polarization balance during injury repair [140]. Third, regarding the impact on fibrotic progression, the activation of fibroblasts and subsequent scar formation are essential processes for cardiac repair after myocardial infarction. Whether ferroptosis participates in regulating fibroblast fate and scar quality remains unclear [141]. Inappropriate intervention might suppress beneficial reparative fibrosis or, conversely, promote pathological fibrosis. Based on this complexity, future intervention strategies should shift from “complete inhibition” to “precise modulation”: finely tuning the level of ferroptosis according to the different stages post-myocardial infarction. This involves moderately inhibiting excessive ferroptosis in the acute phase to salvage cardiomyocytes, while allowing or even promoting necessary ferroptosis in the subacute and chronic phases to facilitate debridement and immune remodeling. In this process, the value of molecular imaging technology will be irreplaceable. It can not only guide “when to initiate” intervention but also, through dynamic monitoring of ferroptosis levels, indicate “when to stop” or adjust the intervention, thereby enabling truly personalized and precise treatment.

## 8. Conclusions

EVs exhibit significant potential for myocardial infarction diagnosis and treatment, attributed to their intrinsic targeting ability, low immunogenicity, and favorable biocompatibility. Playing a particularly important role in regulating cardiomyocyte ferroptosis, exosomes can deliver either pro-ferroptotic signals to aggravate injury or inhibitory molecules to confer protection, supporting their application as targeted therapeutic carriers. Molecular imaging techniques, including MRI, PET/CT, and optical imaging, have enabled the in vivo tracking and efficacy assessment of exosomes, facilitating the real-time visualization of therapeutic processes. To advance the field, future efforts should focus on establishing standardized preparation and imaging databases, as well as developing innovative engineering strategies, ultimately positioning exosomes as a versatile platform for precision theranostics in myocardial infarction.

## Figures and Tables

**Table 1:** The Diagnostic Value of Ferroptosis-Related Genes in AMI.

Gene	Expression	Diagnostic Value	References
*TLR4*	Up	Potential biomarker; associated with immune infiltration; AUC = 0.829	[28]
*PIK3CA*	Down	High diagnostic accuracy; AUC = 0.734	[29]
*miR-216a*	Up	Upregulated in AMI; negatively correlates with *PIK3CA*; potential ceRNA biomarker	[29]
*ACSL1*	Up	High diagnostic value; AUC = 0.873	[30]
*ATG7*	Up	Moderate diagnostic value; AUC = 0.731	[31]
*CAMKK2*	Up	Moderate diagnostic value; AUC = 0.712	[32]
*SLC2A3*	Up	High-risk factor; involved in glucose metabolism and hypoxia response	[33]
*EPAS1*	Up	High-risk factor; regulates hypoxia-inducible pathways	[34]
*HMOX1*	Up	High-risk factor; key enzyme in heme degradation and oxidative stress	[35]
*ATM*	Down	Highly protective factor; DNA damage repair gene	[36]
*FANCD2*	Down	Highly protective factor; involved in DNA repair and genomic stability	[36]

**Table 2:** Comparison of EV imaging modalities.

Technology	Resolution	Sensitivity	Penetration Depth	Typical Applications
Fluorescence Imaging	High (μm level)	Medium (nM level)	<1 cm	Cell/Small animal surface imaging
Bioluminescence Imaging (BLI)	Medium (mm level)	Ultra-high (pM level)	Several centimeters	In vivo dynamic tracking
MRI	Ultra-high (μm level)	Low (mM level)	Unlimited	Anatomical and functional imaging
SPECT	Low (cm level)	Medium (nM level)	Whole body	Multi-target molecular imaging
PET	Medium (mm level)	Ultra-high (nM level)	Whole body	Metabolism and receptor imaging

Abbreviations: BLI, bioluminescence imaging; cm, centimeter; EV, extracellular vesicle; mm, millimeter; mM, millimolar; MRI, magnetic resonance imaging; μm, micrometer; nM, nanomolar; pM, picomolar; PET, positron emission tomography; SPECT, single-photon emission computed tomography.

**Table 3:** In vivo imaging of EVs.

Labeling Method	Type	Specific Materials/Tools	Mechanism	Advantages	Disadvantages	References
Quantum Dots (QDs)	Direct	Semiconductor QDs (e.g., CdSe, CdTe)	Nanoparticles attach to EV membranes or are encapsulated into EV cores via surface modification	High photostability, multicolor labeling, resistance to photobleaching	Potential toxicity, may interfere with native EV functions	[116,117]
Lipophilic Dyes	Direct	PKH67, PKH26, DiR, DiD, DiI	Lipid-soluble dyes insert into EV membrane bilayers; fluorescence increases upon membrane binding	Simple operation, visualization of membrane structure	Dye aggregation, residual signals interfere with long-term tracking	[118,119,120]
MRI Contrast Agents	Direct	Superparamagnetic iron oxide (SPIO)	SPIO loaded into EVs via electroporation, generating T2-weighted MRI signals	High tissue penetration, clinical compatibility	Electroporation may damage EV membranes, residual signals	[121]
Radionuclides	Direct	^99^mTc, ^111^In, Fluorine-18 (^18^F), Copper-64 (^64^Cu)	Covalent binding, membrane labeling, or encapsulation of radioactive isotopes	Ultra-high sensitivity (picomolar level), suitable for whole-body dynamic imaging	Radiation exposure, limited half-life (e.g., ^18^F: 109 min)	[122,123,124]
Fluorescent Proteins	Indirect	GFP, tdTomato (e.g., PalmGFP, PalmtdTomato)	Genetically engineered donor cells express membrane-anchored fluorescent proteins (via palmitoylation)	High specificity, long-term tracking of intercellular EV transfer	Limited tissue penetration (<2 mm), requires fluorescence imaging equipment	[125,126]
Luciferases	Indirect	Fluc (Firefly luciferase), Rluc (Renilla luciferase), Gluc (Gaussia luciferase), NanoLuc	Donor cells express luciferase-CD63 fusion proteins to catalyze substrate luminescence	No background noise, real-time dynamic monitoring, high sensitivity	Requires exogenous substrate injection, signal depends on enzyme activity	[127]
USPIO Labeling	Indirect	Ultrasmall superparamagnetic iron oxide (USPIO)	USPIO pre-loaded into donor cells, released via MVB to form USPIO-EVs	Avoids direct labeling damage, MRI-compatible	Requires prolonged cell incubation, may reduce EV yield	[128]
Membrane Fusion Proteins	Indirect	VSVG5 (viral glycoprotein), CD63-luciferase fusion proteins	Genetic engineering enables EV membrane expression of fusion proteins (e.g., CD63-NanoLuc)	Strong targeting, enables tracking of specific cell types	Depends on transfection efficiency, may alternative EV membrane properties	[128,129]

Abbreviations: EV, extracellular vesicle; Fluc, firefly luciferase; GFP, green fluorescent protein; Gluc, Gaussia luciferase; MRI, magnetic resonance imaging; MVB, multivesicular body; NanoLuc, nanoluciferase; Rluc, Renilla luciferase; SPIO, superparamagnetic iron oxide; tdTomato, tandem dimer Tomato; USPIO, ultrasmall superparamagnetic iron oxide. Direct labeling refers to the covalent or strong physical conjugation of the imaging probe (e.g., radioisotope, fluorophore) directly onto the targeting vector (e.g., antibody, peptide). This approach is simple but may potentially alter the bioactivity of the vector. Indirect labeling involves a two-step (or multi-step) process, where the targeting vector is first modified with an affinity tag or reactive handle (e.g., biotin, click-chemistry group, or enzyme tag), followed by subsequent binding or reaction with the imaging probe. This strategy offers greater flexibility and often better preservation of target binding affinity.

## Data Availability

Data sharing is not applicable to this article as no new data were created or analyzed in this study.

## Abbreviations

AMI	Acute myocardial infarction
EVs	Extracellular vesicles
ROS	Reactive oxygen species
TF	Transferrin
TFR1	Transferrin receptor protein 1
FTH	Ferritin
DMT1	Divalent Metal Transporter 1
HMGB1	High mobility group protein B1
IRPs	Iron regulatory proteins
GPX4	Glutathione peroxidase 4
GSH	Glutathione
SLC7A11	Solute carrier family 7 member 11
L-OOHs	Lipid hydroperoxides
L-Ohs	Lipid alcohols
PUFAs	Polyunsaturated fatty acids
ACSL4	Acyl-CoA Synthetase Long-Chain Family Member 4
4-HNE	4-hydroxynonenal
MDA	Malondialdehyde
MIRI	Myocardial ischemia–reperfusion injury
TIBC	Total iron binding capacity
SF	Serum ferritin
Fer-1	Ferrostatin-1
NAC	N-acetylcysteine
DFO	Deferoxamine
Lip-1	Lipoxstatin-1
SOD1	Superoxide Dismutase 1
MVBs	Multivesicular bodies
SPION	Superparamagnetic Iron Oxide Nanoparticles
STEAP	Six-transmembrane Epithelial Antigen of the Prostate
PEG	Polyethylene glycol
MSCs	Mesenchymal stem cells
CPB	Cardiac bypass grafting
ODC1	Ornithine decarboxylase 1
APCs	Antigen-presenting cells
BLI	Bioluminescence Imaging
PET	Positron emission tomography
SPECT	Single-photon emission computed tomography
MRI	Magnetic resonance imaging
MS	Microspheres
QDs	Quantum dots
CTP	Cardiac targeting peptides
EF	Ejection fraction
USPIO	Ultra-small superparamagnetic iron oxide
MNPs	Magnetic nanoparticles
NVs	Nanocapsules
MFRNs	Ethylene glycol-modified iron oxide nanocubes
GMNPs	Glycol magnetic nanoparticles
QSM	Quantitative Susceptibility Mapping
MPI	Magnetic Particle Imaging
GNPs	Gold nanoparticles
